# Implementation of an “Opt-Out” Tobacco Treatment Program in Six Hospitals in South Carolina

**DOI:** 10.21203/rs.3.rs-3318088/v1

**Published:** 2023-09-08

**Authors:** K. Michael Cummings, Vincent Talbot, Avery Roberson, Asia A. Bliss, Emily Likins, Naomi C. Brownstein, Stephanie Stansell, Demetress Adams-Ludd, Bridget Harris, David Louder, Edward McCutcheon, Rami Zebian, Alana Rojewski, Benjamin A. Toll

**Affiliations:** Medical University of South Carolina; TelASK Inc.; Medical University of South Carolina; Medical University of South Carolina; University of Pikeville; Medical University of South Carolina; Medical University of South Carolina; Medical University of South Carolina; Medical University of South Carolina; Medical University of South Carolina; Medical University of South Carolina; Medical University of South Carolina; Medical University of South Carolina; Medical University of South Carolina

**Keywords:** Cigarette Smoking, Tobacco Cessation, Hospital, Health Services Research, Nicotine Addiction, Population Health

## Abstract

**Objective:**

To describe the implementation an opt-out tobacco treatment program (TTP) in 6 diverse hospitals located in different regions of South Carolina.

**Methods:**

Between March 8, 2021 and December 17, 2021, adult patients (≥ 18 years) admitted to 6 hospitals affiliated with the Medical University of South Carolina (MUSC) were screened for their cigarette status. Patients who smoked cigarettes were referred to an TTP offering a brief bedside consult and automated post-discharge follow-up calls with an opportunity to receive a referral to the South Carolina Quitline (SCQL). The hospitals included in this study ranged in size from 82 to 715 beds with diverse patient populations. Herein, we report on the results of screening and referring patients to the TTP, delivery of smoking cessation treatments, and patient smoking status assessed in a sample of patients followed 6-weeks after discharge from the hospital.

**Results:**

Smoking prevalence ranged from 14–49% across the 6 hospitals. Among eligible patients reached, 85.6% accepted the bedside consult. Only 3.4% of patients reached were deemed ineligible because they claimed not to be currently smoking cigarettes. The automated post-discharge follow-up calls were answered by 43% of patients, with about a third of those who had relapsed back to smoking accepting the offer of a referral to the SCQL. Overall, about half of the 6,000 patients referred to the TTP received some type of treatment. Self-reported smoking abstinence rates assessed 6-weeks after discharge were similar across the five acute care hospitals ranging from about 20–30%.

**Conclusion:**

The findings demonstrate the broad reach of implementing an opt-out TTP for patients in hospitals of varying size, rurality and patient populations.

## Purpose and Objectives

Hospitalizations provide opportunities to identify and engage patients who smoke cigarettes to stop smoking (1–5). We and others have previously provided evidence demonstrating the feasibility and effectiveness of implementing an opt-out inpatient Tobacco Treatment Program (TTP) modeled after the Joint Commission’s (JC) guidelines (5–15). In our earlier evaluation studies, we found the TTP increased post-discharge quit rates, reduced unplanned hospital readmissions, and was cost-effective (14–17). However, our prior studies were limited to data on patients from a single acute care hospital. Since 2016, we have expanded our TTP to include psychiatric inpatient patients and four community hospitals that were recently acquired by the Medical University of South Carolina (MUSC).

In this paper, we report on the results of screening and referring patients to the TTP in 6 MUSC affiliated inpatient hospitals with diverse patient populations from different geographic regions of South Carolina. The University Medical Center in downtown Charleston, South Carolina is comprised of 4 hospitals: Institute of Psychiatry, and three acute care hospitals, Children’s Hospital, Ashley River Tower, and University Hospital. For the purposes of this study, we have combined the latter 3 acute care facilities in Charleston into a single group for reporting purposes, referred to as Charleston Non-Institute of Psychiatry (Non-IOP). The 6 hospitals we report on in this paper include: 1) Charleston Non-IOP with approximately 715 beds; 2) Charleston IOP, which has 105 beds; 3) Florence Medical Center, which has 396 beds; 4) Marion Medical Center, which has 124 beds; 5) Lancaster Medical Center, which has 225 beds; and 6) Chester Medical Center, which has 82 beds. Herein, we report on the results of screening and referring patients to the TTP, delivery of smoking cessation treatments, and patient smoking status assessed in a sample of patients followed 6-weeks after discharge from the hospital.

## Intervention Approach

The MUSC inpatient TTP has been described in detail elsewhere (14). Briefly, the workflow includes 4-steps: 1) screening to identify adult patients who currently smoke cigarettes; 2) referral of patients to the TTP; 3) treatment service delivery; and 4) follow-up evaluation.

In step 1 (screening), all hospitalized patients are asked about whether they currently smoke cigarettes. Smoking status is recorded in the electronic health record (EHR), and all hospitals in this study used Epic^™^.

In step 2 (referral), all adults who self-reported current smoking were referred to the TTP on a daily basis with the following exceptions: a) patient unable to communicate due to language or medical condition; b) patient receiving hospice care; and c) patients who had previously been admitted to the hospital and referred to the TTP in the past 6-months. The 6-month referral criteria were put into place to maximize limited resources directed toward enrolling those patients newly identified as currently smoking.

In step 3 (treatment service delivery), patients referred to the TTP could receive a bedside consult from an ATTUD trained (https://www.attud.org/program-accreditation) tobacco treatment specialist (referred to as “specialist”) and/or automated post-discharge follow-up call offering patients the opportunity for referral to the South Carolina Quit Line (SCQL). During the reference period, we conducted a quality improvement study to evaluate the impact of the bedside consult intervention with and without the post-discharge automated follow-up call. In each hospital setting, approximately 72% of the TTP-eligible patients were randomly assigned to the bedside consult group plus post-discharge calls while the remaining 28% received only the post-discharge automated follow-up calls. Three full-time specialists provided bedside consults to eligible patients. One specialist was assigned to see eligible patients in the two Charleston Medical Center hospitals, a second specialist was assigned to see patients in the Florence and Marion Medical Center hospitals, and the third specialist was assigned to see patients in Lancaster and Chester Medical Center hospitals. The bedside consult utilized motivational interviewing to engage with patients. Patients were able to opt-out of the bedside consult if they wanted. Consult dispositions were categorized as follows: 1) received consult; 2) refused consult; 3) not eligible for consult because the patient reported not smoking currently (i.e., false positive based on intake screening); and 4) unavailable for consult when visited in the hospital. Among patients receiving consults, we recorded whether the consult was conducted in-person or by phone and the length of the consult coded as ≤ 10 minutes or > 10 minutes. Phone consults were utilized when patients were in isolation rooms where in-person visits were restricted and on rare occasions when the specialist could not reach the patient in person prior to their discharge from the hospital. Patients receiving the consult were asked to confirm their smoking status and answer questions about their smoking history, current use of other tobacco products, nicotine dependence, past quit attempts, readiness, and confidence in stopping smoking, all of which were used to create a personalized quitting plan. Specialists worked with hospital providers to dispense nicotine replacement therapy to eligible and interested patients.

Patients who provided a working phone number and were discharged to their home or to assisted living facilities were eligible to receive automated post-discharge follow-up calls starting seven days after hospital discharge. The follow-up call asked patients if they currently smoked cigarettes; those answering “yes” were asked if they wanted to be transferred to the SCQL where they could get free stop smoking assistance. Those who answered “no” to an immediate “warm” transfer were asked if they would like the SCQL to call them instead; those asking for assistance an electronic referral was sent to the SCQL. Up to 6 call attempts were made to reach each patient seven days after discharge from the hospital. If a patient failed to answer any of the calls, then we attempted to reach them again a week later (i.e., 14 days after discharge). For patients eligible to receive automated post-discharge calls, we report on the number who answered the call, the number of current smokers who accepted a referral to the SCQL, and among those referred to the SCQL, the number who received services from the SCQL.

In step 4 (follow-up evaluation), we attempted to contact patients referred to the TTP six-weeks after discharge from the hospital to assess their satisfaction with the care they received while hospitalized, their smoking status, and efforts made to refrain from and/or stop smoking during and following hospitalization. Follow-up evaluation calls were only made to patients with working telephone numbers who were discharged home. Staff conducting follow-up interviews were not involved in providing treatment to patients. To encourage participation in the follow-up evaluation survey, we sent a letter to eligible patients explaining that they had been selected to participate in a brief 10-minute phone survey asking them about their recent hospitalization and alerting them to expect a call within 10 days. Patients who participated in the survey were given a $10 Amazon e-gift card to compensate them for their time. The follow-up evaluation survey excluded psychiatric patients, because the quality improvement study that paid for the 6-week follow-up evaluation survey was limited to patients receiving care in the five acute care hospitals only.

## Evaluation methods

### Data Sources

Data reported in this paper come from: (1) hospital EHRs; (2) the bedside consult electronic form which captured information about the patients smoking history and treatment plan; and (3) the six-week phone follow-up evaluation survey conducted using REDCap. The quality improvement study was reviewed by and received ethics clearance through the Medical University of South Carolina IRB Committee (IRB#107000) with a HIPAA Waiver of Authorization of Research.

EHRs contain information on the patients admission and discharge dates, unit where the patient was located during their hospital stay, demographics (age, race, sex, type of insurance), smoking status (i.e., never, former, current, unknown), and identifying information such as full name, address, and phone number. Data on daily admissions were extracted from EHRs and deposited via a secure, HIPAA-compliant transfer to the Quit Plan Manager^™^ system (QPM 5.0, by TelASK Inc, Mount Pleasant, South Carolina, USA) which identified currently smoking patients eligible for the TTP. Each morning, the list of inpatient smokers from the prior days admissions was presented to the specialist through the QPM secure web-interface that could be accessed using a desktop computer and a tablet. The interface loaded patients’ identifying information to a task list that informs the specialist where patients were located in the hospital. For patients seen by the specialist, information on the consult was captured on the tablet and securely uploaded to the patient database system. Data from the EHRs received daily also allow the patient database system to detect patients discharged from the hospital and eligibility for receiving automated follow-up calls. Based on the patients’ hospital-assigned medical record number, QPM identified patients who have been readmitted to the hospital within the previous six-months and excluded these patients from re-enrollment in the service. Patients were eligible for re-enrollment in the TTP if readmitted to the hospital after 6-months. In this paper, we only report on the patients’ first admission to the hospital during the reference period. Data from EHRs, bedside consult, and automated calls were captured in separate databases and linked together for analysis via the patients’ medical record number and hospital admission date.

Data on all patients enrolled in the TTP allowed the specialists and clinical chief of the TTP (BAT) to track the status of patients eligible for the service. Weekly reports were generated to track screening, referral, and treatment delivery results for each hospital. The six-week post-discharge follow-up evaluation surveys were tracked separately to ensure sufficient interviews were completed for patients in each of the participating hospitals involved in the quality improvement study.

### Data Analysis

Six quality indicators were measured: (1) the proportion screened who endorsed current smoking; (2) among patients reporting current smoking, the proportion of patients eligible for referral(s) to the TTP; (3) among the patients eligible for the bedside consult the proportion who received the consult; (4) among the patients discharged to their home, the proportion who received and answered the automated follow-up call and reported being a current smoker; (5) among current smokers who answered the automated call the proportion who accepted a referral to the SCQL; and (6) among patients who completed the follow-up interview, the proportion who reported not smoking at the time of the 6-week follow-up evaluation. Smoking status was defined among: a) not smoking on the day of the follow-up interview (24-hour point prevalence abstinence); b) not smoking in the past 7-days (7-day point prevalence abstinence); and c) not smoking in the past 30-days (30-day point prevalence abstinence). For patients who report smoking at the time of the six-week follow-up interview, we also report on the proportion who have made a quit attempt since their hospital stay and the proportion interested in receiving additional assistance to stop smoking.

Frequencies and percentages were reported for categorical variables whereas medians, ranges, means, and standard deviations (SD) were reported for continuously measured variables. All statistical analyses were performed in SAS 9.4^™^ (SAS Institute, Cary, NC).

## Results

### Screening

**Figure 1:**
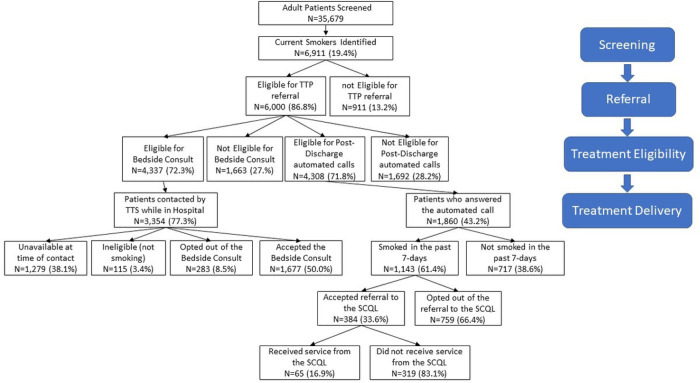
Screening, referral, treatment eligibility, and treatment delivery to hospitalize patients across six MUSC affiliated hospital March 8, 2021 to December 17, 2021.

Between March 8th and December 17th, 2021, there were a total of 47,946 adult admission records identified across the six hospitals representing 35,679 unique patients, of whom 19.4% (6,911/35,679) were currently smoking. Figure 1 provides a flow diagram summarizing the screening, referral, treatment eligibility and treatment delivery across all six hospitals combined. Overall, about half of the 6,000 patients referred to the TTP received some type of treatment with 1,072 (17.9%) receiving the bedside consult only, 1,255 (20.9%) responding to the automated post-discharge call only, and 605 (10.1%) receiving both the bedside consult and automated post-discharge call.

Supplemental tables 1–3, reports the screening, referral and treatment delivery results in each hospital separately. Cigarette smoking prevalence varied significantly across the 6 hospitals with the highest smoking prevalence rate in patients admitted to the psychiatric hospital in Charleston (49.3%) and the lowest rate among patients seen in the acute care hospital in Charleston (13.9%).

Of the 6,000 patients referred to the TTP during the reference period, 4,337 (72.3%) were eligible to receive a bedside consult, while 4,308 patients were eligible for the automated post-discharge follow-up calls offering a referral to the SCQL. Overall, specialist were able to reach 77% (n = 3,354/4,308) of patients eligible for the bedside consult with half (n = 1,677) of the patients reached actually receiving a bedside consult. Consult delivery varied significantly by hospital (see supplemental table 3) with a lower percentage of consults provide to eligible patients in the two Charleston hospitals compared to the four regional hospitals. Overall, most patients approached for the consult accept it (i.e., 85.6%). The proportion of patients who self-reported not smoking was low (n = 115, 3.4%).

Table 1 summarizes the characteristics of patients who received the bedside consult overall and for each hospital. Most of the bedside consults were done in-person, although in one hospital 71% of consults were done by phone. About half of the consults were completed in less than 10 minutes, although the time spent counseling differed among the three specialists. The specialist serving patients in the Florence and Marion Medical Center hospitals reporting over 94% of the consults lasting ≤ 10 minutes, while the other two specialists had the majority of their consults lasting > 10 minutes.

The majority of patients who received a bedside consult were male, smoked cigarettes daily, with an average age of 50 years old or greater. Psychiatric inpatients were younger (mean age: 38.5 years) and reported higher cigarette dependence as assessed by cigarettes per day and time to first cigarette of the day. Among those who smoked cigarettes daily, the average number of cigarettes smoked per day was about 14 cigs/day. About 75% of patients reported having made a previous attempt to stop smoking. Among those reporting a previous quit attempt, cold-turkey was the most common method reported. About 23% of patients reported past use of stop smoking medications, and only 3% mentioned using an e-cigarette to stop smoking. About 35% of patients reported having strong cravings to smoke while in the hospital, although this varied widely by hospital (i.e., 17–62%). Just under 28% of patients reported using stop smoking medications while hospitalized, with wide variations seen across the six hospitals (10–67%). The highest rate of stop smoking medication used while hospitalized was among psychiatric patients where nicotine replacement is offered as a standard of care for daily smokers admitted to the hospital. Readiness and confidence to quit varied across the six hospitals, with about 55% of patients overall contemplating quitting. In terms of treatment planning, most patients had stop smoking medications recommended or provided to them. Although nearly 38% of patients refused stop smoking medication, the exception was psychiatric patients who were either already using or open to using stop medications.

Of the 4,308 patients eligible to receive automated calls, 1,860 patients (43.2%) were reached yielding an overall response rate of 43%. Response rates to the automated calls ranged from 22–53% across the six hospitals (see supplemental table 3). Among those responding to the automated calls, 1,143 (61.5%) reported currently smoking in the past 7-days, and of these patients, 384 (33.6%) accepted the offer of a referral to the SCQL. Data received back from the SCQL found of the 384 patients referred to the SCQL 16.9% received services from the SCQL. There were different reasons given for failure to provide service to the referred patients with the most common reasons being inability to reach patients with follow-up calls (45.2%), patients not interested in the service (19.4%), and 35.4% deemed ineligible because they had been enrolled in the SCQL within the past year.

### Six-Week Follow-up Evaluation Survey

Of the patients from the five acute care hospitals referred to the TTP, 2,957 were selected to participate in the 6-week follow-up evaluation survey. Of those selected to be surveyed, 553 (18.7%) were ineligible for the survey, leaving 2,279 eligible survey respondents. Of eligible survey respondents, 986 were reached and completed the survey, yielding a response rate of 43.3%. Response rates were similar from patients across the five hospitals.

Table 2 summarizes responses to questions about smoking status, the use of stop smoking medications, quit attempts, and interest in receiving additional quitting assistance among those who reported that they still smoke. Self-reported smoking abstinence rates were fairly similar across the 5 hospitals with 21.0% reporting not smoking since discharge from the hospital (continuous abstinence), 30.8% not smoking on the day of the follow-up survey (point prevalence), 24.2% reporting not smoking in the past 7-days (7-day period prevalence), and 22.3% reporting not smoking in the past 30-days (30-day period prevalence). Overall, 40.9% of patients reported getting stop smoking medications either during their hospital stay, at the time of discharge from the hospital, or after discharge. Getting stop smoking medications during their hospital stay varied across the 5 hospitals. In general, the smaller hospitals seemed to have greater success obtaining stop smoking medications to eligible patients during their hospital stay. Among patients still smoking at the time of the follow-up interview (n = 682), 57.5% reported having made a quit attempt since being discharged from the hospital and 43.3% reported interest in receiving assistance to stop smoking.

## Discussion

The United States Department of Health and Human Services recently requested information on strategies to broaden the delivery of evidence-based smoking cessation treatments to those who continue to smoke cigarettes (18). Previous studies have demonstrated the effectiveness of an opt-out smoking cessation treatment model for hospitalized patients (5–15). This study is unique in that it examines practical feasibility of implementing an opt-out smoking cessation treatment model across multiple hospitals serving diverse patient populations. In general, the findings from this study demonstrate the broad reach of implementing an opt-out TTP for hospitalized patients consistent with the JC standards for treating tobacco dependence.

The majority of the patient population referred to the TTP smoked cigarettes daily, and many had tried to quit in the past but had not previously used evidence-based cessation treatments. Among eligible patients reached while in the hospital, 85.6% accepted the bedside consult; 14.4% opted out. These findings suggest that patient resistance to tobacco treatment support is not a significant barrier to treatment delivery. The biggest barrier we experienced in delivering the bedside consult to patients was reaching patients while they were in the hospital. Patients were often too ill to be seen for a consult and/or discharged from the hospital before a consult could be offered. About 40% of patients were not available for a consult when the specialist stopped by the patients hospital room. Failure to deliver the bedside consult was more common in the two Charleston hospitals, reflecting the larger number of patients to be reach along with the unique challenges associated with delivering the bedside consults to psychiatric inpatients. Based on our experience implementing the bedside consults, now in multiple hospitals with diverse patient populations, we estimate that one specialist can handle about 42 new patient referrals per week or about 2,200 patient referrals per year on average.

The automated calls to patients discharged home represents a low-cost way to extend smoking cessation services to patients. Over 40% of patients were reached with the automated call system using interactive voice recognition with about one-third of patients who reported currently smoking accepting a referral to the SCQL. Psychiatric inpatients had a lower response to the automated calls, although among those reached, about 25% accepted the referral to the SCQL. The actual provision of cessation support to patients connected to the SCQL was lower than we had hoped for, and many patients did not answer the follow-up calls or indicated lack of interest in the service after the referral was made, possibly reflecting the patients motivation to quit.

However, the opportunity to reengage with patients after discharge from the hospital offers an opportunity to try to boost patient motivation for smoking cessation. Among patients who reported that they were still smoking in our 6-week follow-up survey, 43% expressed interest in receiving additional cessation assistance, reinforcing the benefits of using the hospital admission and immediate post-discharge period as an opportunity to support patients in their journey towards smoking cessation. Finding more effective ways to link patients who return to smoking after hospital discharge to services that might help them stop smoking is an area that is ripe for future investigations.

Smoking is a chronic relapsing disorder, so even though patients were not allowed to smoke while hospitalized, among those who responded to our automated post-discharge call 7–14 days after being released from the hospital, 61.5% reported that they had smoked a cigarette in the past week. At six-weeks post-charge, self-reported smoking abstinence rates ranged from 20–30% depending on how the abstinence rate was defined. Overall, smoking abstinence rates were similar to what we previously reported in our prior study of an opt-out inpatient TTP and were similar across the five acute care hospitals involved with the quality improvement study (14). While many patients did report getting stop smoking medications during or immediately after discharge from the hospital, the rate of medication use was lower than we had expected it to be, especially among patients receiving the bedside consult where one of the objectives of the consult was to develop and execute a stop smoking treatment plan for patients. The use of stop smoking medications during or after hospitalization was about 40% higher among those who received the bedside consult compared to those who did not (50.6% versus 35.6%), suggesting that recommended treatment plans did have an impact. However, the overall use of stop smoking medications was lower than we had expected given that increasing the use of evidence-based medications was an important component of the TTP. A quality improvement study is currently underway to study the effectiveness of an intervention to increase prescribing of stop smoking medications when patients are admitted and discharged from the hospital.

Like many real-world implementation studies, getting planned treatment services to patients can be challenging. Overall, we were successful in getting treatments delivered to just under half of the referred patients. Because of our quality improvement study, only 72% of the patients referred to the TTP were eligible for the bedside consult, which reduced the overall percentage of patients receiving the bedside consult. That said, it is not realistic to assume delivery of services to all patients. Our sense is that with adequate staffing, delivering of stop smoking services might be achievable for about 60–70% of the total population of patients.

This dissemination implementation analysis also has identified several areas where delivery of the TTP can be improved upon. Most notably, getting stop smoking medications prescribed for patients when discharged from the hospital and improving the system for connecting patients to treatment services after discharge from the hospital needs improvement. Another weakness of this study is our exclusion of psychiatric inpatients from the follow-up evaluation survey. This exclusion was done because of lack of funding to support the follow-up evaluation. However, our data supports the critical need to address smoking in patients with mental health and other substance use disorders. The smoking prevalence was 48% among our psychiatric inpatients. These patients also smoked more heavily compared to patients seen in the acute care hospitals. However, even among patients seen in acute care hospitals, it is likely that many of these patients also have mental health and other substance use problems that are ignored in the context of an intervention with a singular focus on smoking cessation. A recent analysis of patients who currently smoke cigarettes from one of our acute care hospitals (i.e., not a psychiatric hospital) found that 34% had a diagnosis of a mental health or substance use disorder (19).

The annual budget for the inpatient TTP implemented in 6-hospitals was $360,152. The TTP budget includes expenses for three full-time specialist and clinical oversight, training and equipment for the specialists, and the vendor contract for the Quit Plan Manager^™^ system. With a total of 6,000 patients referred to the TTP over 284 days of the reference period the average annual number of referrals is estimated to be 7,709 yielding average annual per patient cost was $47 per referral to the TTP (i.e., $360,152/7,709 = $47).

## Conclusion

In summary, the findings demonstrate the broad reach and practical feasibility of implementing an opt-out tobacco-cessation service for patients seen in hospitals across different geographical regions of varying size and patient populations at a relatively low cost. Overall, patient acceptance of tobacco treatment support was high, although we found elements of our service delivery can be improved upon through better patient-to-staffing ratios, improved systems to get stop smoking medications prescribed for patients, and ways to improve linking patients to stop smoking services after hospital discharge. Also, while a brief hospital-based opt-out intervention is effective and capable of reaching a large proportion of hospital patients, it is important to consider subgroups for patients, such as those with mental health and other substance use disorders, who may need more intensive treatment support to assist them in stopping smoking.

## Figures and Tables

**Table 1.** Characteristics of patients who received the bedside consult

Treatment components	Charleston Non-IOP	Charleston IOP	Florence	Marion	Lancaster	Chester	Combined

Received consult	**472**	**58**	**672**	**111**	**289**	**75**	**1,677**

Encounter							
• In-person	469(99.4%)	58(100%)	635(94.5%)	93(83.8%)	169(58.5%)	22(29.3%)	1,446(86.2%)
• Phone	1(0.2%)	0(0.0%)	37(5.5%)	18(16.2%)	120(41.5%)	53(70.7%)	229(13.7%)
• Missing	2 (0.4%)	0 (0.0%)	0 (0.0%)	0 (0.0%)	0 (0.0%)	0 (0.0%)	2 (0.1%)

Length of consult							
• ≤10 mins	83(17.6%)	23(39.7%)	641(95.4%)	104(93.7%)	6(2.1%)	0(0.0%)	857(51.1%)
• >10 mins	375(79.4%)	33(56.9%)	29(4.3%)	7(6.3%)	282(97.6%)	74(98.7%)	800(47.7%)
• Missing	14 (3.0%)	2 (3.4%)	2 (0.3%)	0 (0.0%)	1 (0.3%)	1 (1.3%)	20 (1.2%)

Mean age years	51.3	38.5	51.3	54.7	52.5	58.2	54.7

Gender							
• Female	193 (40.9%)	26 (44.8%)	304 (45.2%)	55 (49.5%)	151 (52.2%)	39(52.0%)	768 (45.8%)
• Male	279 (59.1%)	32 (55.2%)	368 (54.8%)	56 (50.5%)	138 (47.8%)	36(48.0%)	909 (54.2%)

Product type							
• Cigs only	410(86.9%)	44 75.9%)	579(86.2%)	96 (86.5%)	269(93.1%)	73(97.3%)	1,471(87.7%)
• Cigs & other combustible	25 (5.3%)	5 (8.6%)	59 (8.8%)	7 (6.3%)	12 (4.2%)	2 (2.7%)	110 (6.6%)
• Cigs & non-combustible	27 (5.7%)	6 (10.3%)	33 (4.9%)	7 (6.3%)	8 (2.8%)	0 (0.0%)	81 (4.8%)
• Cigars only	1 (0.2%)	0 (0.0%)	1 (0.1%)	1 (0.9%)	0 (0.0%)	0 (0.0%)	3 (0.2%)
• Oral only	1 (0.2%)	0 (0.0%)	0 (0.0%)	0 (0.0%)	0 (0.0%)	0 (0.0%)	1 (0.1%)
• E-cigs only	0 (0.0%)	1 (1.7%)	0 (0.0%)	0 (0.0%)	0 (0.0%)	0 (0.0%)	1 (0.1%)
• Missing	8 (1.7%)	2 (6.4%)	0 (0.0%)	0 (0.0%)	0 (0.0%)	0 (0.0%)	10 (0.6%)

Mean cig duration years	27.3	19.2	29.4	31.4	31.1	34.8	29.1

Cig use							
• Daily	409(86.7%)	51(87.9%)	584(86.9%)	94(84.7%)	254(87.9%)	63(84.0%)	1,455(86.8%)
• Non-daily	44(9.3%)	4(6.9%)	63(9.4%)	12(10.8%)	34(11.8%)	10(13.3%)	167(10.0%)
• Missing	19(4.0%)	3(5.2%)	25(3.7%)	5(4.5%)	1(0.3%)	2(2.7%)	55(3.3%)

Cigs per day*	13.5	20.1	13.2	14.4	14.5	11.6	13.8

Time to 1^st^ cig							
• ≤5 mins	213(45.1%)	37(63.8%)	216(32.1%)	31 (27.9%)	252(87.2%)	66(88.0%)	815(48.6%)
• 6–30 mins	81(17.2%)	8(13.8%)	140(20.8%)	32(28.8%)	28(9.7%)	7(9.3%)	296(17.7%)
• 31–60 mins	30 (6.4%)	3 (5.2%)	99 (14.7%)	11 (9.9%)	1 (0.4%)	0 (0.0%)	144 (8.6%)
• >60 mins	115 (24.4%)	6 (10.3%)	176 (26.2%)	30 (27.0%)	5 (1.7%)	2 (2.7%)	334 (19.9%)
• Missing/NA	33 (7.0%)	4 (6.9%)	41 (6.1%)	7 (6.3%)	3 (1.0%)	0 (0.0%)	88 (5.3%)

Past quit attempts							
• None	127(26.9%)	14(24.1%)	150(22.3%)	25 (22.5%)	78 (27.0%)	21 (28.0%)	415 (24.8%)
• 1	194(41.1%)	23(39.7%)	231(34.4%)	39 (35.1%)	97 (33.6%)	19(25.3%)	603 (36.0%)
• >1	127(26.8%)	16(27.6%)	276(41.1%)	41 (36.9%)	112(38.8%)	34(45.3%)	606 (36.1%)
• Missing	24 (5.1%)	5 (8.6%)	15 (2.2%)	6 (5.4%)	2 (0.7%)	1 (1.3%)	53 (3.2%)

past quit methods**							
• Nothing	254(79.1%)	36(92.3%)	345(68.0%)	54(67.5%)	138(66.0%)	37(69.8%)	864(71.5%)
• Meds	45(14.1%)	2(5.1%)	129(25.4%)	21(26.3%)	65(31.1%)	15(28.3%)	275(22.8%)
• E-cig only)	14 (4.4%)	1 (2.6%)	16 (3.2%)	2 (2.5%)	4 (1.9%)	0 (0.0%)	36 (3.0%)
• Quitline or classes	2 (0.6%)	0 (0.0%)	8 (1.6%)	2 (2.5%)	0 (0.0%)	0 (0.0%)	12 (1.0%)
• Missing	5 (1.6%)	0 (0.0%)	9 (1.8%)	1 (1.3%)	2 (1.0%)	1 (1.9%)	21 (1.7%)

Cravings							
• Yes	224(47.5%)	36(62.1%)	179(26.6%)	19(17.1%)	109(37.7%)	28(37.3%)	595(35.5%)
• No	229(48.5%)	19(32.8%)	463(68.9%)	84(75.7%)	179(61.9%)	47(62.7%)	1021(60.9%)
• Missing	19 (4.0%)	3 (5.2%)	30 (4.5%)	8 (7.2%)	1 (0.4%)	0 (0.0%)	61 (3.6%)

Currently use meds	46(9.8%)	39(67.2%)	154(22.9%)	60(54.1%)	142(49.1%)	25(33.3%)	466(27.8%)

Mean confidence to quit score***	7.1	5.9	4.3	4.4	5.8	6.3	5.4

Stage of change							
• No interest	110(23.3%)	20(34.5%)	143(21.3%)	21(18.9%)	67(23.2%)	18(24.0%)	379(22.6%)
• Not ready, cut down	59(12.5%)	3(5.2%)	49(7.3%)	6(5.4%)	93(32.2%)	22(29.3%)	232(13.8%)
• Interested in quitting	263(55.7%)	29(50.0%)	425(63.2%)	74(66.7%)	110(38.1%)	26(34.7%)	927(55.3%)
• Already quit	25 (5.3%)	3 (5.2%)	16 (2.4%)	0 (0.0%)	17 (5.9%)	8 (10.7%)	69 (4.1%)
• Missing	15 (3.2%)	3 (5.2%)	39 (5.8%)	10 (9.0%)	2 (0.7%)	1 (1.3%)	70 (4.2%)

Cessation meds							
• None	9(1.9%)	0(0.0%)	120(17.9%)	11(9.9%)	11(3.8%)	0(0.0%)	151(9.0%)
• Refused	161(34.1%)	4 (6.9%)	291(43.3%)	40(36.0%)	103(35.6%)	31(41.3%)	630(37.6%)
• Recommend	160(33.9%)	20(34.5%)	102(15.2%)	13(11.7%)	16(5.5%)	4(5.3%)	315(18.8%)
• Provided	131(27.6%)	32(55.2%)	156(23.2%)	46(41.4%)	159(55.0%)	39(52.0%)	563 (33.6%)
• Missing	11 (2.3%)	2 (3.4%)	3 (0.5%)	1 (0.9%)	0 (0.0%)	1 (1.3%)	18 (1.1%)

* Cigs per day is computed for daily smokers

** Reported quit methods is computed based on those who reported one or more quit attempts

*** Confidence rating is rated on a scale ranging from 0 (no confidence) to 10 (high confidence)

**Table 2:** Six-week post-discharge follow-up evaluation survey outcomes

Smoking related outcomes	Charleston Non-IOP N = 495	Florence N = 251	Marion N = 65	Lancaster N = 134	Chester N = 41	Combined N = 986

Continuous abstinence	108 (21.8%)	53 (21.1%)	14 (21.5%)	25 (18.7)	7 (17.1%)	207 (21.0%)

Point prevalence abstinence	147 (29.7%)	87 (34.7%)	22 (33.9%)	39 (29.1%)	9 (22.0%)	304 (30.8%)

7-day period prevalence abstinence	124 (25.1%)	63 (25.1%)	15 (23.1%)	29 (21.6%)	8 (19.5%)	239 (24.2%)

30-day period prevalence abstinence	113 (22.9%)	57 (22.7%)	14 (21.5%)	28 (20.9%)	8/41 (19.5%)	220 (22.3%)

Made a quit attempt*	203 (58.3%)	93 (56.7%)	23 (53.5%)	54 (56.8%)	19 (59.4%)	392 (57.5%)

Interest in quit assistance*	140 (40.2%)	74 (45.1%)	22 (51.2%)	45 (47.4%)	14 (43.8%)	295 (43.3%)

Received medications						
• In hospital	165 (33.3%)	79 (31.5%)	35 (53.8%)	71 (53.3%)	20 (48.8%)	370 (37.6%)
• At discharge	95 (19.2%)	38 (15.1%)	15 (23.1%)	26 (19.3%)	5 (12.2%)	179 (18.1%)
• After discharge	125 (25.3%)	56 (22.3%)	16 (24.6%)	40 (29.6%)	12 (29.3%)	249 (24.2%)
• Any meds	186 (36.8%)	91 (36.3%)	32 (49.2%)	79 (59.0%)	19 (46.3%)	403 (40.9%)

Satisfied with care received in the hospital	475 (96.0%)	235 (93.6%)	58 (89.2%)	128 (94.8%)	39 (95.1%)	935 (94.7%)

* Restricted to those who answered “Yes” to having smoked today.

## Data Availability

A de-identified dataset, data dictionary and copy of our REDCap survey are available upon request made to lead author (K. Michael Cummings, cummingk@musc.edu).

## Abbreviations

TTP: Tobacco Treatment Program
SCQL: South Carolina Quitline
JC: Joint Commission
EHR: Electronic Health Record
ATTUD: Association for the Treatment of Tobacco Use and Dependence
Specialist: Tobacco Treatment Specialist
